# The influence of Nav1.9 channels on intestinal hyperpathia and dysmotility

**DOI:** 10.1080/19336950.2023.2212350

**Published:** 2023-05-15

**Authors:** Chenyu Zhao, Xi Zhou, Xiaoliu Shi

**Affiliations:** a Department of Gastroenterology, Henan Provincial People’s Hospital, Zhengzhou University People’s Hospital, Zhengzhou, China; b Department of Gastroenterology, The Second Xiangya Hospital, Central South University, Changsha, China; c Department of Medical Genetics, The Second Xiangya Hospital, Central South University, Changsha, China; d The National & Local Joint Engineering Laboratory of Animal Peptide Drug Development, College of Life Sciences, Hunan Normal University, Changsha, Hunan, China

**Keywords:** Nav1.9 voltage-gated sodium channels, *SCN11A*, intestinal motility, visceral pain, irritable bowel syndrome

## Abstract

The Nav1.9 channel is a voltage-gated sodium channel. It plays a vital role in the generation of pain and the formation of neuronal hyperexcitability after inflammation. It is highly expressed in small diameter neurons of dorsal root ganglions and Dogiel II neurons in enteric nervous system. The small diameter neurons in dorsal root ganglions are the primary sensory neurons of pain conduction. Nav1.9 channels also participate in regulating intestinal motility. Functional enhancements of Nav1.9 channels to a certain extent lead to hyperexcitability of small diameter dorsal root ganglion neurons. The hyperexcitability of the neurons can cause visceral hyperalgesia. Intestinofugal afferent neurons and intrinsic primary afferent neurons in enteric nervous system belong to Dogiel type II neurons. Their excitability can also be regulated by Nav1.9 channels. The hyperexcitability of intestinofugal afferent neurons abnormally activate entero-enteric inhibitory reflexes. The hyperexcitability of intrinsic primary afferent neurons disturb peristaltic waves by abnormally activating peristaltic reflexes. This review discusses the role of Nav1.9 channels in intestinal hyperpathia and dysmotility.

## Introduction

The Nav1.9 channel is a type of voltage-gated sodium channel (VGSC), that can regulate the resting membrane potential and amplify subthreshold stimuli [1]. The α subunit encoded by the *SCN11A* gene is the main functional subunit of Nav1.9 channels [2]. Nav1.9 channels are highly expressed in membranes of small diameter afferent neurons in dorsal root ganglions (DRGs) and trigeminal ganglions, and Dogiel II neurons in the enteric nervous system (ENS) [3,4]. In the past, Nav1.9 channels were mainly considered to be involved in the formation of pain sensing [5]. The small-diameter neurons of the DRG are the primary neurons involved in pain transduction. The pathogenic variations of the *SCN11A* gene lead to three genetic pain related disorders: familial episodic pain type III (FEP type III), congenital insensitive to pain (CIP), and genetic small fiber neuropathy (SFN) [6,7]. Some patients carrying the pathogenic variants in the *SCN11A* gene exhibit not only abnormal pain, but also gastrointestinal symptoms [8–11]. Recently, Nav1.9 channels have also been found to participate in regulating colonic motility [12]. Intestinal hyperpathia and dysmotility are vital physiopathologic mechanisms in certain functional gastrointestinal disorders (FGIDs), such as irritable bowel syndrome (IBS) [13,14]. This review addresses the role of Nav1.9 channels in intestinal hyperpathia and dysmotility. Its underlying mechanisms are explained.

## Structure and function of Nav1.9 channels

**Figure 1.**
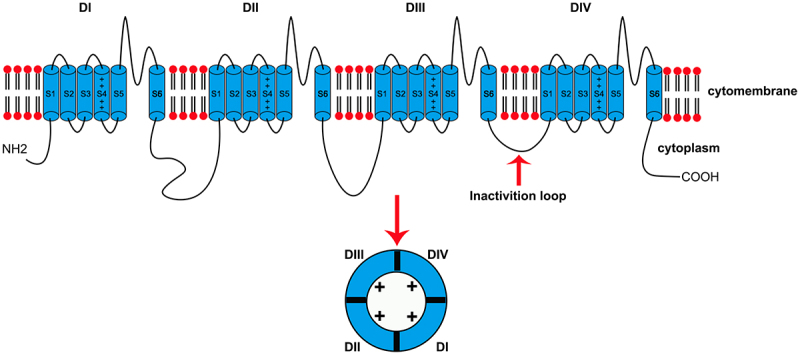
Schematic diagram of the VGSC structure. VGSCs comprise 4 transmembrane domains (DI-IV). Each domain consists of 6 transmembrane segments (S1–6). S1–4 comprise the voltage sensing domain. There are positively charged amino acid residues in S4 on every 3 amino acid residues. The interlinked peptide chain between S5 and S6 is called the pore loop (P loop). The P loops of the four domains form the sodium ion selective filter. The S5-S6 and P loops form the pore domain. The intracellular inactivation motif between DIII and DIV can block the channel. There is a tetrodotoxin binding site on the channels. The 4 domains form a pore inserted through the cell membrane. D, domain; S, segment; TTX, tetrodotoxin; +, positive charge. This figure was modified from Coates [3].

The Nav1.9 channel consists of α and β subunits. The α subunit is the main functional subunit and forms ion-selective channels. The β subunit is the coregulatory subunit. A total of 10 sodium channels have been found in mammals, including 9 VGSCs. According to the α subunit, VGSCs are named Nav1.1–1.9 channels. The molecular weight of the α subunit is approximately 260 kDa, and there is only one α subunit per channel. The α subunit contains four homologous transmembrane domains (DI-DIV), which are linearly arranged in the same chain. Each domain contains six transmembrane α helical segments (S1-S6) [15]. S1-S4 comprise the voltage sensing domain (VSD). The interlinked peptide chain between S5 and S6 is called the pore loop (P loop). The P loops of the four domains form the sodium ion selective filter. The S5-S6 and P loop form the pore domain. In the VSD, there is a positively charged amino acid residue (arginine or lysine) in S4 located on every 3 amino acid residues. Inactivation motifs (IFMs) containing isoleucine, phenylalanine, and methionine are found in the intracellular ring between the DIII and DIV domains. The IFM can block the channel and participate in inactivation of the channel when the membrane depolarizes continuously. There are drug and toxin binding sites on the α subunit [16]. β subunits modulate the activation and inactivation kinetics of VGSCs, regulate channel density at the membrane, and may also have a role in regulating channel subcellular localization [1,17] (Figure 1). The expression of Nav1.9 channels peaks in adolescence and declines significantly after the beginning of adulthood [7].

**Figure 2.**
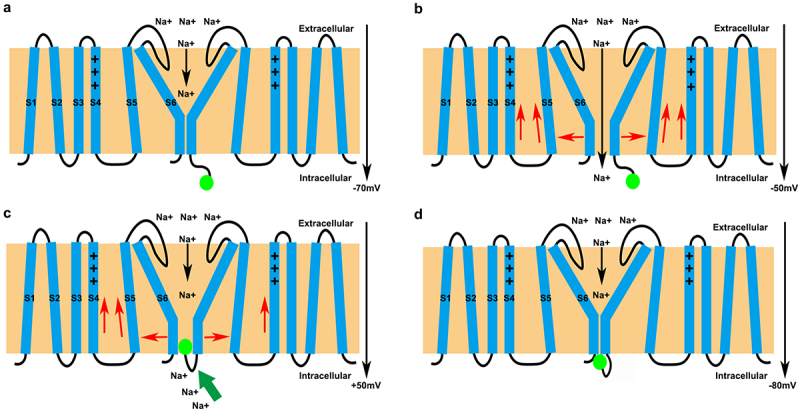
The gating mechanisms of VGSCs. (a) the cell membrane is at the resting potential. The pore is closed, and the IFM is in the resting conformation. (b) the depolarization above the threshold potential makes S4 move outwards and the pore open. Sodium ions flow into the cell. (c) the changes in potential after pore opening cause the IFM to block the open pore. (d) Activity of other ion channels produces a hyperpolarized membrane potential, causing S4 to return to its resting position. In addition, the pore closed. This figure was modified from Bennett [7].

The depolarization of the cell membrane potential induces the activation and opening of VGSCs. It selectively mediates transmembrane sodium influx, and is one of the molecular bases of cell action potential generation. The gating mechanisms of VGSCs can be summarized as a “two-gate three-state model” [3]. VGSCs have an activation gate and an inactivation gate, and three states, including resting (standby), activation and inactivation states. When the cell membrane is at resting potentials, the activation gate of VGSCs closes, and the inactivation gate opens. VGSCs could respond to the electrical signal depolarization stimulus. When cell membrane depolarization reaches the activation threshold, the position of S4 could shift, leading to conformational changes in the α subunit. The activation and inactivation gates of VGSCs both open, temporarily allowing sodium ions to enter cells. Then, in the fast inactivation mode, the activation gate opens. However, the inactivation gate closes. The IFM blocks the channel area. Later, other ion channels are activated to generate a hyperpolarized membrane potential (approximately −80 mV), which causes S4 to return to its resting position. Both the activation and inactivation gates close, which is referred to as slow inactivation. In the inactive state, VGSCs cannot be activated again. With the repolarization of the membrane potential, the channel revives and returns to its resting state [7] (Figure 2).

The α subunit of the Nav1.9 channel is encoded by the *SCN11A* gene (OMIM: * 604385). At present, the Human Gene Mutation Database (HGMD) contains information regarding 32 pathogenic mutations of the *SCN11A* gene. Human Nav1.9 channels contain 1791 amino acid residues and are classified as tetrodotoxin-resistant (TTX-R) channels in pharmacology [18]. TTX is a potent neurotoxin that blocks VGSCs. TTX binding to the α-subunit within the outer vestibule of the VGSC in a highly selective manner, blocking the entry of Na^+^ ions through the channel. VGSCs are classified on the basis of its sensitivity to TTX. TTX-R VGSCs are inhibited at micromolar concentrations of TTX. However, TTX-sensitive VGSCs are blocked by nanomolar concentrations. TTX-sensitive VGSCs include Nav1.1, Nav1.2, Nav1.3, Nav1.4, Nav1.6 and Nav1.7 channels. Nav1.5, Nav1.8 and Nav1.9 channels belong to TTX-R VGSCs [19]. The electrophysiological characteristics of Nav1.9 channels are relatively unique. Its activation voltage is very low and is close to the resting membrane potential of the neuronal membrane (−70 mV ~ −40 mV). In addition, the inactivation of Nav1.9 channels is ultraslow, with steady-state activation and inactivation overlapping. Nav1.9 channels can open spontaneously and continuously at voltages within a specific overlap range, generating a large window current [20]. Thus, Nav1.9 channels act as threshold channels to regulate resting membrane potentials by generating inward sodium currents. In addition, Nav1.9 channels respond to subthreshold stimulations [20,21], which reduces the discharge threshold of a single action potential and increases the number of repeated neuronal discharges [22].

Heterologous expression of Nav1.9 channels was difficult previously, which limited the screening of Nav1.9 channels modulators [23]. Recently HEK-293 cells and ND7/23 cells that heterologously express Nav1.9 channels were established [24,25]. They can facilitate identification of selective Nav1.9 modulators. There are spider venom-derived peptides specifically activating Nav1.9 channels [26,27]. The leech peptide HSTX-I exerts significant analgesic function by specifically inhibiting Nav1.8 and 1.9 channels [28]. However, another study suggested that HSTX-I was not a useful lead for development of analgesic Nav1.8 and 1.9 modulators [29]. The specific inhibitors of Nav1.9 channels need further study.

## The role of Nav1.9 channels in intestinal hyperpathia

### Nav1.9 channels and pain production

**Figure 3.**
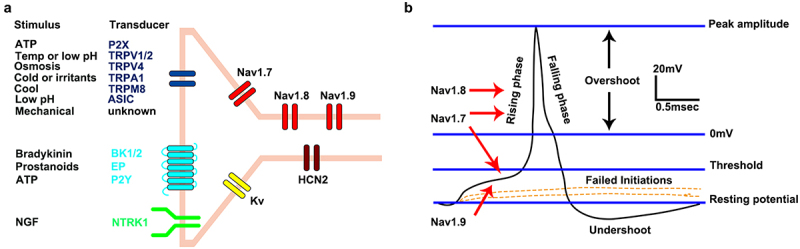
The generation of action potentials in nociceptor neurons. (a) These neurons transduce signals regarding extreme temperatures (Temp), low pH, and various chemical stimuli into electrical signals by some of the ligand-gated ion channels (blue), G-protein-coupled receptors (light blue), and tyrosine kinase receptors (green). Then, VGSCs, such as Nav1.7, Nav1.8 and Nav1.9 (red), have a key role in responding to small depolarizations and action-potential generation. The hyperpolarizing activated cation channel HCN2 acts as a so-called pacemaker, modulating ectopic activity after nerve injury. Voltage-gated potassium channels (yellow) are important breaks in excitability. This figure was cited from Bennett [7]. (b) Representative action potential waveform recorded from a small-diameter nociceptive DRG neuron, excited by a depolarizing current step of 200 pA. The contributions of the Nav1.7, Nav1.8 and Nav1.9 channels to the generation of the action potential are indicated. This figure was modified from Bennett [1].

The process of pain generation is complex. The endings of nociceptive sensory neurons express some ligand-gated ion channels, G-protein-coupled receptors and tyrosine kinase receptors. Through these receptors, nociceptive neurons transmit signals regarding extreme temperature, low pH, and various chemical stimuli into electrical signals. VGSCs play a key role in the response to small depolarization potentials and action potential generation [7] (Figure 3a). Then, neural impulses are transmitted to the dorsal horn of the spinal cord, which is the primary integration center for pain signal processing in the trunk and limbs. Neural impulses are gradually relayed to the cerebral cortex through the spinal cord. The cerebral cortex produces pain. Hyperalgesia indicates a decreased pain threshold for stimuli. Allodynia is a condition in which pain is triggered by stimuli that are not supposed to cause pain.

The DRG is an oval enlargement of the spinal nerve at the intervertebral foramen, which is also known as the sensory ganglia. The DRG is formed from the aggregated cell bodies of pseudomonopolar neurons in vertebrates. The free ends of the process of these cells are used as receptors, which are distributed in the skin, joints, muscles and internal organs. The central process of DRG neurons projects nerve impulses to the posterior horn of the spinal cord. The sensory neurons contained in the DRG are important signal integration and conduction centers in sensory pathways, such as those for pain, temperature and pressure sensations. Small-diameter neurons in the DRG are primary neurons involved in pain transduction [30]. DRG neurons can be divided into large-, medium- and small-diameter neurons. Large-diameter neurons mainly emit Aα and Aβ fibers with coarse myelin. Medium-diameter neurons mainly emit Aδ fibers with fine myelin. Small neurons mainly emit C fibers without myelin [1]. Nav1.9 channels are mainly expressed in small-diameter DRG neurons [31,32], but also in medium- and large-diameter neurons [33]. Nav1.9 channels are distributed on the cell membranes of the cell bodies, central processes and peripheral processes of these primary afferent neurons [5,34,35]. During the action potential production of small-diameter DRG neurons, the Nav1.9 channel is responsible for amplifying subthreshold stimuli. The Nav1.7 channel is responsible for amplifying subthreshold stimuli and is involved in the formation of action potential ramus. The Nav1.8 channel is the main VGSC responsible for the formation of the action potential rising phase [1] (Figure 3b).

The Nav1.9 channel is involved in the production of inflammatory pain [36]. First, inflammatory factors, such as bradykinin, histamine and prostaglandin E2, can directly or indirectly upregulate the activity of Nav1.9 channels [37]. Inflammation reduces local cholesterol levels. The decrease in cholesterol levels can sensitize nociceptive neurons and promote hyperalgesia [38]. In addition, the inflammatory response upregulates the expression of Nav1.9 channels in DRG sensory neurons [39].

Both Nav1.7 and Nav1.9 are involved in the summation of subthreshold stimuli and determining the threshold for action potential generation. Pathogenic mutations in Nav1.7 channels cause several pain related disorders in human, including congenital insensitivity to pain, inherited erythromelalgia, paroxysmal extreme pain disorder and inherited small-fiber neuropathy [1]. Patients with mutations in Nav1.7 channels do not manifest the same intestinal complications that are observed in patients with mutations in Nav1.9 channels. Nav1.7 is mainly abundantly expressed in vagal sensory neurons and DRG sensory neurons [1]. Therefore, mutant Nav1.7 channels have limited effect on ENS. These patients are not affected with obvious intestinal dysmotility. But Nav1.7 channels can influence gut sensation. For example, paroxysmal extreme pain disorder is originally known as familial rectal pain [40].

### *Pain-related genetic diseases associated with* SCN11A *gene variation*

Pathogenic *SCN11A* variants can lead to three different genetic diseases: FEP type III, genetic SFN and CIP [5]. FEP type III is also called familial episodic limb pain, which exhibits clinical features of paroxysmal limb pain. It is a rare autosomal dominant genetic disease. Its incidence in the population has not been reported. Only individual cases have been reported at present [8,9,41–44]. Gain-of-function *SCN11A* mutations cause FEP type III. Mutant Nav1.9 channels in patients with FEP are more likely to open, which results in the hyperexcitability of small-diameter DRG neurons. Then, the hyperexcitability of the neurons ultimately leads to hyperpathia [42].

SFN is also known as painful small fiber neuropathy. Its main clinical manifestations are pain, numbness, burning and other paresthesia. SFN is usually accompanied by autonomic nervous dysfunction, such as hyperhidrosis, and gastrointestinal motility disorders. It can be caused by metabolic diseases, drug, autoimmune, infection, genetics and other causes, which affect the fibers of the peripheral sensory nerves. Gain-of-function mutations in the *SCN11A* gene lead to partial genetic SFN [45–49]. This mechanism can partially explain the pathogenesis of SFN [50,51].

The main clinical characteristics of CIP caused by the *SCN11A* gene are insensitivity to pain, self-injurious behaviors, and slow wound healing. The condition may be accompanied by itching, Charcot arthropathy, muscle weakness and abnormal intestinal motility. It is a rare genetic disease, and most studies are case reports [10,11,52–54]. The obvious gain-of-function *SCN11A* mutations could mediate significantly more sodium influx and directly reach the inactivation potentials of Nav1.7 and Nav1.8 channels. Nav1.7 and Nav1.8 channels cannot mediate sodium influx, which hinders the formation of action potentials, and ultimately leads to insensitivity to pain [55].

Some patients carrying *SCN11A* mutations showed intestinal dysmotility [8–11]. For example, a patient with CIP carrying the L396P variant had constipation [10]. The FEP patient with the R222S variant exhibited diarrhea [41]. In addition, the CIP patient with the L811P variant has alternate symptoms of diarrhea and constipation [11].

### Alterations in the function of Nav1.9 channels and intestinal hyperpathia

*Scn11a* gene knockout (*Scn11a*^−/−^) mice showed no significant changes in visceral nociceptive sensitivity under the noninflammatory state. However, *Scn11a*^−/−^ mice exhibited decreased visceral nociceptive hyperreactivity induced by inflammatory mediators. In electrophysiological tests, the responses of intestinal afferent nerves to pain-causing inflammatory mediators (adenosine triphosphate (ATP) and prostaglandin E2) were significantly lower than those in the wild-type (WT) group [56]. In addition, *Scn11a*^−/−^ and WT mice were tested for the sensitivity to colorectal pain induced by mechanical distension stimuli using the abdominal withdrawal reflex (AWR) test. There was no significant difference between the two groups. Then, after the mice were subjected to enema with an inflammatory inducer, WT mice showed higher levels of colorectal pain sensitivity than the placebo group. However, *Scn11a*^−/−^ mice responded similarly to the placebo treatment. This finding suggested that Nav1.9 channels were involved in inflammation-related acute colorectal hyperalgesia [57]. Such afferent fibers, which are normally insensitive to stimuli but are activated in an inflammatory state, are known as silent or “dormant” pain receptors [58].

In contrast, *Scn11a*^R222S/R222S^ mice carried the gain-of-function R222S variant in the *Scn11a* gene, which was homologous to the R222S variant in FEP patients. They showed not only somatic hyperalgesia, but also visceral hyperalgesia. In the AWR test, the *Scn11a*^R222S/R222S^ mice presented with increased pain sensitivity to mechanical stimulation. In addition, the acute inflammatory visceral pain thresholds of *Scn11a*^R222S/R222S^ mice also decreased significantly [59]. Some patients with pathogenic *SCN11A* gene variants also have abdominal pain symptoms [8,9].

### The potential mechanisms underlying the effects of Nav1.9 channels on visceral hyperalgesia

Extrinsic afferent neurons distributed in the gastrointestinal tract include spinal afferent nerves and vagal afferent nerves, both of which innervate the whole gastrointestinal tract. In the upper digestive tract, the main extrinsic afferent nerves come from vagus nerves. In the lower digestive tract, the distribution of vagus nerves gradually decreases, and the number of spinal afferent nerves increase. The vagus nerves are not involved in sensory formation. The main external afferents distributed in the colon are spinal afferents, which have cell bodies that are located in the DRG [60]. Lumbosacral and vertebral DRGs project fibers to the distal colon and bladder along with the pelvic nerves [61,62]. Except for the distal colon, spinal afferent nerves in other parts of the intestinal tract mainly travel along the splanchnic nerves and merge into the thoracic and lumbar DRGs [63].

The small-diameter neurons in the DRG are primary neurons of peripheral pain transduction, which mainly belong to type C nerve fibers [64]. They could be divided into non-peptidergic neurons that could be labeled by IB4 and peptidergic neurons that expressed calcitonin gene related peptide (CGRP). The Nav1.9 channel is mainly expressed in nonpeptidergic DRG neurons (IB4+) [35]. In addition, 14% of sensory neurons distributed in the colon are IB4+ neurons [65]. Single-cell RNA sequencing of DRG neurons revealed that the Nav1.9 channel is expressed in peptidergic neurons, non-peptidergic neurons, myelinated neurons and nonmyelinated neurons [66]. A study showed that Nav1.9 channels are expressed in half of the nerve fibers projected to the intestine by DRG [56]. Gain-of-function changes in Nav1.9 channels to some extent lead to hyperexcitability of small-diameter DRG neurons. Under the circumstance, some subthreshold stimulations can generate nerve impulses in these neurons. Small-diameter DRG neurons are primary neurons for pain signal processing in the trunk and limbs. Therefore, the alterations of Nav1.9 channels could furtherly cause hyperpathia, including visceral hyperalgesia. Small-diameter DRG neurons in *Scn11a*^R222S/R222S^ mice, for instance, showed hyperexcitability. These mice manifested visceral hyperpathia [59].

## The role of Nav1.9 channels in intestinal dysmotility

### The neural regulation of intestinal motility

The ENS is relatively independent of the central nervous system (CNS), also known as the gut brain or the second brain. The ENS has complete internal neural reflex circuits. It can maintain intestinal function, even if the connections with the CNS and autonomic nervous system (ANS) are completely cut off [67]. The ENS comprises the myenteric plexus and the submucosal plexus. The myenteric plexus is mainly responsible for the regulation of intestinal movements. The submucosal plexus is involved in the secretion of water and electrolytes, as well as the neural regulation of intestinal blood flow [68]. The ENS plays a leading role in the neuroregulation of intestinal motility. For example, in one experiment, the exogenous nerve fibers of isolated intestinal segments in guinea pigs were completely cut off. Then, intestinal segments continued to carry out complex neuro-driven propulsive movements in the organ bath by relying on the ENS [69].

Neurons in the ENS are mainly divided into Dogiel type I and Dogiel type II neurons in morphology. The cell bodies of Dogiel type I neurons project multiple short dendrites and a single elongated axon. Dogiel type II neurons have smooth cell bodies and project multiple axons, but no dendrites or only a few dendrites [70]. In addition, ENS neurons are divided into sensory neurons, interneurons and motor neurons in terms of functional characteristics [71], and AH-type neurons and S-type neurons in terms of electrophysiological characteristics [72]. AH-type neurons are characterized by the larger action potentials with an infection on the falling phase and long afterhyperpolarizing potential (AHP;>2 s) that follows. S-type neurons are typified by the brief action potentials without slow AHPs, and they present fast excitatory postsynaptic potentials (EPSPs) [68]. The intrinsic sensory neurons/intrinsic primary afferent neurons (IPANs) of the ENS generally have the characteristics of Dogiel II neurons in morphology, and belong to AH neurons in electrophysiological characteristics [67,73,74]. However, some Dogiel type I neurons (intermediate neurons) can function as mechanosensory neurons [75,76]. Nav1.9 channels are mainly expressed in AH-type/Dogiel type II neurons in the ENS [4,77,78]. Nav1.9 channels flow at subthreshold voltages, generating tonic firing. They remain active during the falling phase, opposes action potential repolarization [4].

The gastrointestinal tract displays a wide range of complex motor patterns. Two of the major neurogenic contractile behaviors are segmentation contractions [79,80] and peristalsis [81–83]. Peristaltic reflexes are the basis of most intestinal propulsive movements [84]. However, the identities of the neural circuits participating in other motility patterns remain elusive. How the gut transitions between different motility patterns requires further investigation [68].

Dogiel type II neurons play an important role in intestinal peristaltic reflexes [60]. In peristaltic reflexes, AH neurons/IPANs are activated by mechanical pulling on the intestinal wall and/or chemical stimulation of intestinal contents. After generating nerve impulses, IPANs transmit excitatory signals to both ascending and descending interneurons by releasing substance P (SP), acetylcholine (Ach), glutamate and calcitonin gene-related peptide (CGRP). Then, ascending interneurons release SP and Ach to the excitatory motor neurons on the oral side. The excitatory motor neurons release SP and Ach at the neuromuscular junction, causing smooth muscle contraction. At the same time, the descending interneurons release 5-HT and Ach to the inhibitory motor neurons in the anal side. Then, the inhibitory motor neurons release nitric oxide (NO), vasoactive intestinal polypeptide (VIP) and ATP to the neuromuscular junction, causing the smooth muscle to relax [68,85].

The extrinsic neuromodulation of intestinal motility mainly depends on the ANS. The main neurotransmitter released by sympathetic nerves at synapses is noradrenaline (NE). NE relaxes the smooth muscles of the intestine and inhibits peristalsis. Ach is the main neurotransmitter released by the parasympathetic nerve at synapses. Ach can make intestinal smooth muscle contract and promote intestinal motility [86]. Intestinofugal afferent neurons (IFANs) are a special type of afferent neuron in the ENS. In other words, there are four types of intestinal afferent neurons: IPANs, IFANs, primary afferent neurons with cell bodies located in the DRG and primary afferent neurons with cell bodies located in the vagal ganglion [87]. IFANs belonging to Dogiel II neurons can project impulses to sympathetic neurons in the prevertebral ganglion (PVG), which in turn innervate the intestine [87,88]. IFANs and sympathetic PVG neurons form the basis of entero-enteric inhibitory reflexes, which do not require CNS involvement [89,90]. When mechanical stimulation of intestinal dilation activates the IFANs, the information is transmitted upward to sympathetic motor neurons in the PVG [91,92], and these sympathetic postganglial nerve fibers project into the intestine and inhibit intestinal motility [60]. For example, when a section of the colon of guinea pigs is stretched and dilated, the external sympathetic reflex pathway can inhibit peristalsis of adjacent isolated colon segments in the organ bath [93].

There are several subtypes of VGSCs expressed in the human ENS, including Nav1.1, Nav1.2, Nav1.3, Nav1.5, Nav1.6, Nav1.7 and Nav1.9 channels. The Nav1.5 and Nav1.9 channels are expressed in enteric neurons of mice [63]. Nav1.9 channels are expressed in the intermuscular plexus and submucosal plexus [77,94]. The Nav1.9 channel is mainly distributed in Dogiel II neurons, while the Nav1.5 channel is highly expressed in Dogiel I and Dogiel II neurons in mice. Nav1.9 channels are responsible for amplifying subliminal stimuli and regulating the firing threshold of action potentials in Dogiel II neurons in the ENS, while Nav1.5 channels open to mediate sodium influx and form the ascending branch of action potentials in mice [4,78]. In other words, the Nav1.9 channel is expressed in the ENS, and in small-diameter neurons of the DRG that are associated with the ENS and CNS. Therefore, the functional and structural changes in Nav1.9 channels have a structural basis for affecting intestinal function.

### Alterations in the function of Nav1.9 channels and intestinal dysmotility

Loss-of-function alterations in Nav1.9 channels promote intestinal motility in mice, whereas gain-of-function changes in Nav1.9 channels inhibit intestinal motility in mice. In one study, the migrating motor complex in the isolated colon of *Scn11a*^−/−^ mice was examined. The whole colon was taken and fixed in an organ bath filled with Kirschner’s fluid. The mechanical activity of the circumferential muscles in the proximal, middle and distal parts of the WT and *Scn11a*^−/−^ mice was recorded. The mean frequency and area under the contraction curve in the *Scn11a*^−/−^ mice were significantly higher than those in WT mice [12]. This finding suggested that loss-of-function alterations in Nav1.9 channels promote intestinal motility.

However, a CIP patient with a gain-of-function L811P mutation was observed to exhibit reduced intestinal motility during laparoscopic surgery [11]. We summarized the functional effects of *SCN11A* variants on electronical properties, pain sensing, and potential roles in intestinal hyperpathia and dysmotility (Table 1).

**Table 1.** Summarization of SCN11A variants.

*SCN11A* variants	Diseases	Gastrointestinal symptoms	Effects on properties of Nav1.9 channels	Reference
c.95C>T p.A32V	SFN	NA	NA	[48]
c.151C>T p.R51W	SFN	NA	NA	[48]
c. 664C>A p. R222S	FEP	Diarrhea	Gain-of-function	[41]
c. 665G>A p. R222H	FEP	Diarrhea	Gain-of-function	[41]
c. 673C>T p. R225C	FEP	/	Gain-of-function	[42]
c.1060C>T p.R354W	SFN	NA	NA	[48]
c. 1142T>C p. I381T	SFN	Diarrhea	Gain-of-function	[45]
c. 1187 *T*> C p. L396P	CIP	Constipation	Gain-of-function	[10]
c. 1257G>C p. K419N	SFN	NA	NA	[45]
c.1442G>A p.G481E	SFN	NA	NA	[49]
c. 1744G>A p. A582T	SFN	NA	NA	[45]
c. 2042C>A p. A681D	SFN	NA	NA	[45]
c. 2095G>A p. G699R	SFN	/	Gain-of-function	[46]
c.2419A>G p.I807V	SFN	NA	NA	[48]
c. 2423C>G p. A808G	FEP	/	Gain-of-function	[42]
c. 2432T>C p. L811P	CIP	Constipation and diarrhea	Gain-of-function	[11]
c. 2441T>G p. F814C	FEP	Constipation	Gain-of-function	[8]
c. 2448T>G p. N816K	FEP	Constipation, abdominal pain	Gain-of-function	[9]
c. 2458A >T p. N820Y	FEP	/	NA	[43]
c. 2513G>A p. R838Q	FEP	/	NA	[44]
c. 2524G>C p. A842P	SFN	NA	NA	[45]
c.3437T>C p.F1146S	FEP	Abdominal pain	Gain-of-function	[8]
c.3473T>C p.L1158P	SFN	Diarrhea	Gain-of-function	[45]
c.3506A>G; p.N1169S	SFN	/	NA	[47]
c.3551T>C p.V1184A	FEP	Constipation	NA	[8]
c.3877A>G p.I1293V	SFN	/	NA	[47]
c.3904C>T p.L1302F	CIP	Diarrhea	NA	[54]
c.3222A>G p.I1074M	SFN	NA	NA	[48]
c.3745G>A p.A1249T	SFN	NA	NA	[48]
c.4057-1G>A	SFN	NA	NA	[45]
c.4049G>A p.R1350Q	SFN	NA	NA	[48]
c.4064G>T p.C1355F	CIP	/	NA	[53]
c.4282G>A p.G1428S	SFN	NA	NA	[48]
c.4309G>A p.V1437M	SFN	NA	NA	[48]
c.4826C>T p.T1609I	SFN	NA	NA	[49]
c.5065T>C p.F1689L	SFN	NA	NA	[45]

Abbreviations: FEP, familial episodic pain; CIP, congenital insensitive to pain; SFN, small fiber neuropathy; NA, not available.

*Scn11a*^+/L799P^ mice carried the mutation that is homologous to the L811P mutation in humans. The intestinal segment of *Scn11a*^+/L799P^ mice showed a slightly decreased spontaneous peristalsis frequency, but there was no statistically significant difference from that observed in the WT mice [95]. The carbon powder propelling test was used to investigate the difference in intestinal motility of *Scn11a*^R222S/R222S^ mice in vivo. The distances of the carbon powder traveled in the *Scn11a*^R222S/R222S^ mice were significantly shorter than those in WT mice. These results suggested that intestinal peristalsis was slower in *Scn11a*^R222S/R222S^ mice. The mechanical activity of small intestine segments was evaluated. The frequency of contractions in intestinal segments from *Scn11a*^R222S/R222S^ mice was lower than that in WT mice [59]. Therefore, the gain-of-function changes in Nav1.9 channels inhibit intestinal motility in mice.

### The potential mechanisms underlying the effects of Nav1.9 channels on intestinal dysmotility

**Figure 4.**
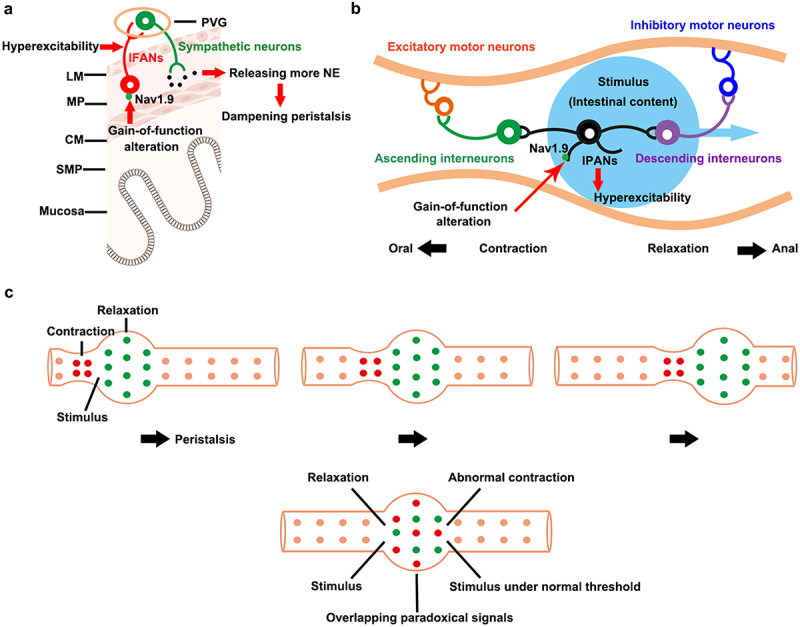
The underlying mechanisms for the hyperexcitability in Nav1.9 channels that causes intestinal dysmotility. (a) the influence on entero-enteric inhibitory reflexes. Gain-of-function alteration in Nav1.9 channels could lead to hyperexcitability of IFANs. The excess stimulation by IFANs might cause sympathetic neurons in PVG releasing more NE. NE could dampen intestinal peristalsis. LM: longitudinal smooth muscle, MP: myenteric plexus, CM: circular smooth muscle, SMP: submucosal plexus, PVG: prevertebral ganglion, IFANs: intestinofugal afferent neurons, NE: noradrenaline. (b) Alteration in peristalsis reflexes. Gain-of-function change in Nav1.9 channels cause hyperexcitability of IPANs. IPANs: intrinsic primary afferent neurons. (c) the abnormal peristalsis reflexes triggered by hyperexcitability IPANs may destroy intestinal peristalsis waves. Overactive firing in IPANs might lead to overlapping paradoxical signals. The mixed signals may disrupt normal pressure gradients in peristalsis. This figure was modified from Zhao [59]. .

Visceral sensory afferent neurons overstimulate motor neurons in the PVG, which can affect local motion of the gastrointestinal tract [14]. *Scn11a*^R222S/R222S^ mice showed a slight increase in intestinal NE concentrations in tissues [59] (Figure 4a). The gain-of-function changes in Nav1.9 channels may lead to hyperexcitable IFAN afferent fibers, which could abnormally activate entero-enteric inhibitory reflexes. The sympathetic motor neurons in the PVG are then stimulated. These sympathetic efferent nerve fibers can then release the neurotransmitter norepinephrine, and inhibit intestinal motility.

In regions of inflammation, IPANs are spontaneously active and synaptic activities are enhanced. These changes result in the overlap of upward excitatory signals and downward inhibitory signals in the inflammatory region. In addition, inhibitory neuromuscular transmission is decreased. When peristaltic waves arrive at the region, the peristalsis is disrupted by the mixed signals and the suppressed neuromuscular transmission [96]. *Scn11a*^R222S/R222S^ mice showed dampened intestinal motility. IPANs in *Scn11a*^R222S/R222S^ mice might exhibit hyperexcitability [59] (Figure 4b). They might be activated by subthreshold stimulations. If abnormal signals triggered by overactive IPANs were contradictory to normal signals in the same area, mixed signals would appear. The mixed signals might disrupt normal pressure gradients during peristalsis (Figure 4c). In addition, the persistent TTX-R Na+ currents mediated by the Nav1.9 channels may be involved in the regulation of neuronal excitability in vagal afferent neurons. The activation of visceral afferent fibers may induce autonomic changes and alterations in colonic tone (e.g. vagally mediated gastrocolonic motor response). Therefore, Nav1.9 channels may affect intestinal motility by autonomic changes. However, the regulation process of gastrointestinal motility is very complex, and the effect of Nav1.9 channels on intestinal motility and its specific mechanisms still need to be further studied. Performing RNA-seq sequencing on intestinal tissue in both *Scn11a* variants knock-in and wild-type mice, for instance, is helpful to investigate potential pathways for this complex network. In order to analyze the electrical activities, it is necessary to isolate enteric neurons from the myenteric plexus of *Scn11a* variants knock-in mice. Then using whole-cell patch clamp technique to check whether IFANs and IPANs manifesting hyperexcitability. Besides, intestinal myoelectrical activities in *Scn11a* variants knock-in mice are worthwhile to research.

## Outlook on the relationship between Nav1.9 channels and IBS

Intestinal hyperalgesia and dysmotility play an important role in the occurrence and development of functional bowel diseases, especially IBS. IBS is a functional bowel disorder characterized by abdominal pain, distention, or abdominal discomfort. It is often accompanied by changes in bowel habits [frequency and/or traits]. Routine clinical examinations typically show no biochemical or morphological abnormalities that could explain these symptoms. The pathophysiological mechanism of IBS has not been fully elucidated, but visceral hypersensitivity (VH) is considered to be the core pathogenic mechanism underlying IBS. VH refers to the enhanced sensitivity of visceral tissues and organs to stimuli, which mainly manifest as hyperalgesia and dysalgesia [97]. Currently, the internationally recognized diagnostic criteria for IBS, the Rome IV criteria, are recurrent abdominal pain with a frequency of more than 1 day/week in the last 3 months as a necessary condition for the diagnosis of IBS [98]. VH occurs in 33–90% of patients with IBS [99]. Moreover, studies have found that visceral and somatosensory thresholds in patients with IBS are both significantly lower than those in healthy controls [100]. Gastrointestinal dysmotility is an important pathophysiological mechanism underlying IBS, and is mainly manifested in intestinal dysmotility [101,102]. In patients with constipation-predominant IBS, the amplitude and frequency of the intestinal migrating motor complex (MMC) are reduced [103], and the intestinal transmission time is also prolonged [101].

IBS following acute gastrointestinal infection is known as postinfectious irritable bowel syndrome (PI-IBS) [104]. IBS was previously considered to be a functional gastrointestinal disease without morphological changes and biochemical abnormalities. However, it is currently believed that chronic low-grade inflammation is involved in the pathogenesis of PI-IBS [105,106]. It is well known that the Nav1.9 channel is normally “dormant” or “silent,” but is involved in inflammatory pain. The expression levels and activities of Nav1.9 channels are upregulated after acute inflammation [37,39]. In addition to acute inflammation, the Nav1.9 channel is involved in hyperalgesia to mechanical and thermal stimuli in mice under subacute and chronic inflammatory states [107]. Therefore, whether the Nav1.9 channel is involved in the pathogenesis of IBS deserves further study.

## Conclusion

Nav1.9 channels play a role in intestinal hyperpathia and dysmotility. The gain-of-function alterations in Nav1.9 channels cause the hyperexcitability of small diameter neurons in the DRG and Dogiel II neurons in the ENS. The hyperexcitability of small -diameter DRG neurons projecting to the intestine leads to intestinal hyperpathia. In addition, the increasing excitability of Dogiel II neurons in the ENS might affect intestinal peristaltic reflexes and entero-enteric inhibitory reflexes, which could cause intestinal dysmotility. VH and gastrointestinal dysmotility are vital pathophysiological mechanisms of IBS. Therefore, functional changes in Nav1.9 channels may be involved in the pathogenesis of IBS, but further studies are needed.

## Data Availability

Data sharing is not applicable to this article as no new data were created or analyzed in this study.
